# Mr. Gilbert's Fulcrum

**Published:** 1850-10

**Authors:** H. Gilbert

**Affiliations:** 5 Suffolk Place, Pall Mall, East, London


### ARTICLE XVI.

### *Mr. Gilbert's Fulcrum.*

Sir,—In your last number of the Provincial Medical and Surgical Journal, you reviewed my pamphlet on the *Extraction of Teeth, with an account of a New and much less Painful Mode of Operating*, but for the following reasons could not recommend it to your readers; had you, however, inspected the fulcrum and chair yourself, or sent some competent person, as did the editors of the other medical and surgical journals of repute, before offering to criticise, I am sure you would have given the same favorable verdict. Allow me, therefore, in justice to myself as the inventor, and the profession and public you have unintentionally misled, to refute your objection. You state:

"To any one who understands the commonest laws of mechanics, it will be at once apparent, that in raising a lower tooth, for instance, out of the socket, as proposed, the jaw is forcibly pressed against the under side of the fulcrum, and the amount of injurious pressure is just as great as it would be if the fulcrum were loose in the mouth, as in the old pelican elevator. Indeed we are inclined to think that the injury may be greater, since in Mr. Gilbert's fulcrum, no adaptation can take place to the surface of the teeth, but some one point must receive all the pressure required, whilst the fulcrum afforded by the pelican, adjusts itself so as to take its bearing from all the available points, and is consequently not so likely to prove injurious, either by damaging the crowns, or producing inflammation of the sockets.

"We are decidely of opinion that in the majority of cases the sound adjacent teeth are exposed to injury from the pressure of *any fulcrum which can be used against a power sufficient to raise the tooth out of the socket*, for it is quite evident that if you lift the tooth, the jaw either goes with it or is prevented doing so by some antagonistic force, and as that force cannot be applied to the soft parts, its application is limited to the teeth themselves, as in both the instrument of Mr. Gilbert and the discarded elevator.

"Should these opinions be practically falsified, no one will rejoice more than ourselves, but after very mature consideration of the subject, we cannot, conscientiously, recommend the apparatus to our readers, although we are fully sensible of the great advantage of the principle, if it could be satisfactorily carried out."

Now, I contend that the principle is carried out by my fulcrum, and that your objection falls to the ground ; for the teeth do not come to any injurious contact with the fulcrum, for the following reasons, which you have entirely overlooked or forgotten :—In extracting a tooth with the forceps with one hand, you place the other on the chin to counteract the antagonistic force, assisted also by the patient's own weight and muscular resistance (if the lower jaw is to be operated upon, as exemplified by you.) No injurious consequences have ever happened. Should the teeth come in contact with the fulcrum, which being covered with some soft material, as gutta percha, no one tooth is particularly pressed upon, as hundreds can testify, on whom I have operated. Many were eminent surgeons, artists, dentists and mechanics, and all are satisfied of the great superiority of my invention. Should you require further evidence, I send the address of a gentleman from whom I extracted a large upper molar, wearing at the same time upper artificial porcelain teeth, which would have broken like glass, had they come in forcible contact with the fulcrum. I know any thing new has many difficulties to contend with, but I trust my great practical experience—moreover, the fact of eminent dentists sending me some of their most difficult cases of extraction, and the testimony of those who daily use my fulcrum and chair, to the exclusion of the old method, must be a further guarantee of the value of the invention.

When exhibiting and explaining the use of my fulcrum, at the meeting of the South-Eastern Branch of our Provincial Medical and Surgical Association, at Brighton, of which I am a member, not an objection was raised, but by all praised, as as did the members of the Medico-Chirurgical Society, Westminster Medical, Medico-Botanical, &c. &c.

Should you have any further doubt on the subject, against such evidence, and against such high medical and surgical authorities as the *Lancet*, *Medical Times*, &c. &c., do come and have a tooth extracted, when I am sure you will go away and exclaim with Mr. Punch—"Go to the admirable Mr. Gilbert, in Suffolk Place, who is dragged into this essay for the benefit of mankind alone, and who, I vow, removes a grinder with so little pain, that all the world should be made aware of him."

I am, Sir,
Your obedient servant,
H. GILBERT.

*5 Suffolk Place, Pall Mall, East,*
London, Septemher 28, 1849.

[We willingly give insertion to the above letter, although we have no inclination to risk the destruction of a valuable tooth for the sake of proving the truth of opinion. We beg, however, to remind Mr. Gilbert, that it was only on his repeated solicitation that we reviewed his pamphlet, thinking the notice in the report of the South-Eastern Branch quite sufficient for the purpose of bringing his invention before the public. We would also remark, that the editor of a journal is not to be led by the opinions expressed by his contemporaries, but rather to judge for himself, which we have endeavored to do in this instance. With regard to Mr. Gilbert's reasons for disputing the position maintained in our review, we regret extremely, that they have not altered the opinion formerly expressed. If the force of the hand on the chin could be sufficient to antagonise the power exerted by the forceps with the assistance of the leverage afforded by the fulcrum, then the use of the instrument cannot be great in raising the tooth perpendicularly from the socket; for we cannot suppose the left hand stronger than the right. The fulcrum, therefore, we think, if of any use, must be strongly depressed, or rather, the jaw must be elevated with great power, and if so, the danger of injury to the sound teeth proportionably increased; and this we hold to be the most important point to guard against in the whole range of dental surgery.—*Ed. Provincial Medical and Surgical Journal.*]

